# Experimental evaluation of *Salmonella* Choleraesuis pathogenicity and porcine reproductive and respiratory syndrome virus synergy in weaned pigs

**DOI:** 10.1186/s40813-026-00502-8

**Published:** 2026-03-11

**Authors:** Eun-Mi Kim, You-Chan Bae, Hyunkyoung Lee, Mi-Hye Hwang, Kyoung-Ki Lee, Hye-Young Jeoung, Go-Eun Shin, Bok-Kyung Ku, Jongho Kim

**Affiliations:** Animal and Plant Quarantine Agency, 177 Hyeoksin 8-ro, Gimcheon, Gyeongbuk 39660 Republic of Korea

**Keywords:** African swine fever, Pathology, Pigs, Porcine reproductive and respiratory syndrome virus, *Salmonella* Choleraesuis, Septicemic salmonellosis

## Abstract

**Background:**

*Salmonella enterica* serovar Choleraesuis (*S.* Choleraesuis) causes septicemic salmonellosis in pigs, which requires differential diagnosis from African swine fever (ASF). Porcine reproductive and respiratory syndrome virus (PRRSV) facilitates *S.* Choleraesuis dissemination. Herein, we determined the pathogenicity of *S.* Choleraesuis and the synergistic effect of PRRSV in septicemic salmonellosis. Furthermore, we compared the pathological characteristics of septicemic salmonellosis and ASF.

**Results:**

Two experimental studies were conducted using weaning pigs (8- and 6-week-old pigs). In the first study, 8-week-old pigs (*n* = 36) were divided into three groups and inoculated with *S.* Choleraesuis (SC group), *Salmonella* Typhimurium (ST group), and phosphate-buffered saline (PBS, control group). Necropsies and *Salmonella* quantification were performed at 1, 3, 7, and 14 days post-inoculation (dpi). *Salmonella* was isolated only from mesenteric lymph nodes in the SC (3–14 dpi) and ST (3 dpi) groups. The SC group showed relatively higher body temperatures, more severe clinical signs, and pronounced histopathological lesions in mesenteric lymph nodes and Peyer’s patches than did the ST group. In the second study, 40 pigs were assigned to four groups: 12 pigs coinfected with *S.* Choleraesuis and PRRSV (PRRSV + SC group), 12 pigs infected with *S.* Choleraesuis (SC group), 8 pigs infected with PRRSV, and 8 pigs inoculated with PBS (control group). They were monitored until 16 dpi and euthanized on 2, 6, 8, and 16 dpi to determine body weight, clinical signs, hematological changes, histopathological examination, and *S.* Choleraesuis colonization. PRRSV + SC pigs had the lowest average daily weight gain and higher mean values, including body temperatures and clinical signs than those of the other groups. They also showed more extensive tissue colonization with *S.* Choleraesuis than that in the SC group. Compared to the principal pathological characteristics of ASF, some pigs in the SC and PRRSV + SC groups showed splenomegaly with dark discoloration and gastrohepatic lymph node enlargement without hemorrhage. However, renal lymph nodes remained unaffected.

**Conclusions:**

Pigs infected with *S.* Choleraesuis revealed higher pathogenicity than did those infected with *S.* Typhimurium, and coinfections with PRRSV enhanced systemic infection. Distinct lesions in gastrohepatic and renal lymph nodes aid differential diagnosis between septicemic salmonellosis and ASF.

**Supplementary Information:**

The online version contains supplementary material available at 10.1186/s40813-026-00502-8.

## Background

Swine salmonellosis, caused by *Salmonella* spp., manifests as septicemia, enterocolitis, or subclinical infection [1]. Septicemia predominantly involves *Salmonella enterica* serovar Choleraesuis (*S.* Choleraesuis), a swine-adapted serovar [1, 2]. *Salmonella enterica* serovar Typhimurium (*S*. Typhimurium), the most prevalent serovar in Korea and the European Union, typically induces enterocolitis but may also result in systemic dissemination and organ-specific lesions in pigs [1–5]. Although salmonellosis affects pigs across all age groups, septicemic salmonellosis caused by *S.* Choleraesuis most frequently occurs in weaned pigs under 5 months of age [2, 4]. The first documented case of septicemic salmonellosis involving *S.* Choleraesuis coinfection with porcine reproductive and respiratory syndrome virus (PRRSV) in Korean weaned pigs was reported in 2022. However, no pathogenicity studies have utilized Korean *S.* Choleraesuis isolates, and limited data exist regarding the impact of PRRSV on the virulence of these isolates [1].

Septicemic salmonellosis warrants consideration in the differential diagnosis of African swine fever virus (ASFV) owing to the similar clinical presentations and gross pathological findings [2]. African swine fever (ASF) has sporadically affected domestic and wild pig populations in Korea since September 2019 and continues to pose a substantial threat to swine health and industry sustainability [6]. In acute and subacute ASF cases, including those observed in Korea, extensive hemorrhagic lesions across multiple organ systems are characteristic. Hemorrhage and edema, in lymphoid tissues, particularly the gastrohepatic and renal lymph nodes, are hallmark gross lesions [6–9].

We conducted experimental inoculations to evaluate the pathogenicity of *S.* Choleraesuis and to elucidate the synergistic effects of PRRSV coinfection in septicemic salmonellosis. Additionally, we performed comparative pathological analysis of lesions, including those in the gastrohepatic lymph nodes, to distinguish septicemic salmonellosis from ASFV infection.

## Methods

### Ethical approval

This study was approved by the Institutional Animal Care and Use Committee of the Animal and Plant Quarantine Agency prior to initiation (approval no. 2022 − 574).

### *Salmonella* inoculum preparation for in vivo studies and animals

The *S.* Choleraesuis strain, which was isolated from a pig with systemic lesions as used in a previous study, was selected for the *S.* Choleraesuis inoculum [1]. The *S*. Typhimurium isolate was obtained from a case of septicemic salmonellosis (characterized by cyanosis, splenomegaly, and multiple petechiae on renal surfaces) and was used to inoculate commercial pigs. These isolates were resistant to streptomycin. A single colony was inoculated into 30 mL of Luria-Bertani broth (Becton Dickinson, Sparks, MD, USA) and incubated for 15 h at 37℃. Bacterial concentration was estimated by measuring optical density at 600 nm (OD600), with a target OD600 of 1 corresponding to approximately 1 × 10⁹ colony-forming units (CFU)/mL. Once the inoculum was prepared, serial dilutions of the inoculum were cultured on 5% sheep blood agar (Asan Pharm. Co., Ltd., Seoul, Korea) to determine the actual concentration of *Salmonella*.

Seventy-six commercial mixed-breed pigs from a single commercial farm were used: 7-week-old pigs (*n* = 36; mean weight ± standard deviation [SD]: 15.7 ± 1.8 kg) and 4-week-old pigs (*n* = 40; mean weight ± SD: 9.0 ± 1.5 kg). The farm of origin, located in Uiseong-gun, Gyeongsangbuk-do, is a farrow-to-finish farm with high biosecurity and hygiene standards and is free from important swine infectious disease pathogens, including ASFV, classical swine fever virus (CSFV), PRRSV, porcine epidemic diarrhea virus (PEDV), and foot-and-mouth disease virus (FMDV). Routine vaccination programs for porcine circovirus 2 (PCV 2), CSFV, FMDV, *Mycoplasma hyopneumoniae*, and *Actinobacillus pleuropneumonia* were conducted. During the 7-day acclimatization period, blood, nasal swabs, and fecal samples were collected from each pig. All pigs were tested negative for antibodies against *Salmonella* spp. in serum using a commercial enzyme-linked immunospecific assay (Swine *Salmonella* Antibody Test kit; IDEXX Laboratories, Westbrook, ME, USA), as well as for nucleic acids in blood, nasal swabs, and fecal samples using various commercial PCR kits for PRRSV (AnyQvet PRRSV qRT-PCR kit, KOREAGENE, Chuncheon, Korea), PCV 2 (PCV2/PCV3 qPCR Custom Kit, KOREAGENE), PEDV (the LiliF TGEV/PEDV RT-PCR Kit, iNtRON Biotechnology InC., Seongnam, Korea), porcine rotavirus (POBGEN™ Rotavirus [A, B, C] Detection Kit, POSTBIO Inc., Namyangju, Korea), and swine influenza virus (SIV qRT-PCR Kit, iNtRON Biotechnology Inc.). To confirm the absence of *Salmonella* spp., 1 g of feces from all pigs was inoculated in 9 mL of Rappaport-Vassiliadis R10 broth (Becton Dickinson) and incubated at 42℃ for 24 h. One loop of Rappaport-Vassiliadis culture was streaked onto CHROMagar *Salmonella* Plus (CHROMagar, Paris, France). *Salmonella* spp. were not isolated from any pigs, and no *Salmonella* spp. were identified from incubated Rappaport–Vassiliadis broths.

**Fig. 1:**
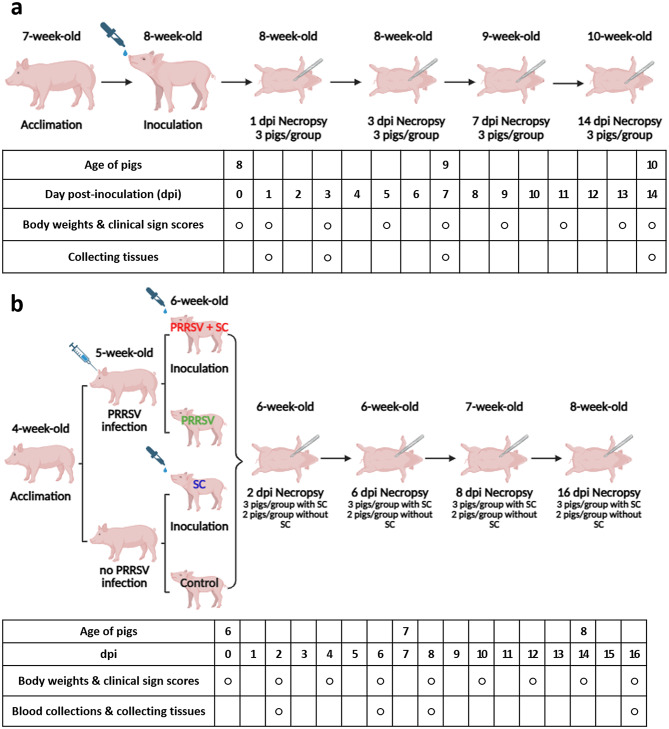
Experimental scheme for design 1 using 8-week-old pigs (**a**) and 2 using 6-week-old pigs (**b**). All pigs in designs 1 and 2 were acclimated for 1 week; circles indicate days on which specific tasks were conducted. (**a**) Pigs in the two groups were orally inoculated with *Salmonella* Choleraesuis (SC, *n* = 12) and *Salmonella* Typhimurium (ST, *n* = 12) (2 × 10^8^ CFU/mL, 10 mL), and pigs in the Control (*n* = 12) group received 10 mL of phosphate-buffered saline (PBS). (**b**) Before the SC inoculation (7 days ago), half of the pigs (*n* = 20) were intramuscularly infected with porcine reproductive and respiratory syndrome virus (PRRSV, 1 × 10^3^ tissue culture infectious dose/mL, 1 mL), and SC inoculations were conducted in 24 pigs with PRRSV (PRRSV + SC, *n* = 12) and without PRRSV (SC, *n* = 12). Other 16 pigs, including PRRSV (*n* = 8) and Control (*n* = 8), received 10 mL of PBS. Images were created with BioRender.com. dpi, days post-inoculation

Body weight, rectal temperature, and clinical signs were monitored every 2 days following *Salmonella* spp. inoculation. The experimental schedule is depicted in Fig. 1. Clinical scores were assigned based on fever, behavior, skin, diarrhea, and body condition, following established criteria (Additional file 1) [10]. We designated experimental groups based on the inoculated pathogens: SC (*S.* Choleraesuis), ST (*S.* Typhimurium), and PRRSV + SC (coinfection with PRRSV and *S*. Choleraesuis).

### Experimental study 1: Comparative analysis of pathogenicity of *S.* Choleraesuis and *S.* Typhimurium in weaning pigs

To determine if the *S.* Choleraesuis isolate has the ability to outcompete other pathogenic and, most prevalent *Salmonella* serovar, *S.* Typhimurium, experimental study 1 was conducted to compare the pathogenicity of *S.* Choleraesuis and *S.* Typhimurium in weaning pigs. Eight-week-old pigs (*n* = 24) were orally inoculated with *S.* Choleraesuis (SC group, *n* = 12; 10 mL of 2 × 10^8^ CFU/mL) or *S.* Typhimurium (ST group, *n* = 12; 10 mL of 2 × 10^8^ CFU/mL). An additional 12 pigs received 10 mL of phosphate-buffered saline (PBS) as controls. The experimental design is illustrated in Fig. 1a. Three pigs in each group (Control, SC, and ST) were euthanized, and postmortem evaluations were performed at 1, 3, 7, and 14 days post-inoculation (dpi). To analyze bacterial loads in organs (brain, lungs, liver, spleen, and mesenteric lymph nodes) and blood, samples were aseptically collected during necropsy. Furthermore, samples of the brain, tonsil, lungs, heart, liver, spleen, kidneys, small and large intestines, and mesenteric lymph nodes were obtained for histopathological examination.

### Experimental study 2: Pathological characterization of *S*. Choleraesuis and PRRSV + *S*. Choleraesuis coinfection in early weaned pigs

To evaluate the pathogenicity and synergistic effects of *S.* Choleraesuis coinfected with PRRSV, 4-week-old early weaned pigs (*n* = 40) were used. PRRSV (PRRSV2, GGYC45 strain, GenBank: MZ287324), genotypically characterized from the previous study, was used to inoculate pigs [11]. The isolate originated from clinically affected pigs with severe bronchointerstitial pneumonia [11]. The virus titers were measured by detecting the 50% tissue culture infected dose (TCID_50_) in MARC-145 cells. In brief, MARC-145 cells were seeded in 96-well cell culture plates and inoculated with a tenfold serial dilution of the supernatant at 37 °C for 2 h. Cells with cytopathic effect were recorded, and the TCID_50_ value was calculated using the Reed and Muench method [12]. The entire experimental process is illustrated in Fig. 1b. After acclimatization, the 5-week-old pigs were randomly divided into four groups (PRRSV + SC, SC, Control, and PRRSV), and 20 pigs (PRRSV + SC and PRRSV) were intramuscularly inoculated with 1** × **10^3^ TCID_50_/mL of PRRSV in 1 mL of PBS. After 7 days, 24 pigs (PRRSV + SC and SC) were orally inoculated with 2** × **10^8^ CFU of *S.* Choleraesuis wild isolate in 10 mL of PBS, and 16 pigs (Control and PRRSV) were orally inoculated with 10 mL of PBS. Three pigs (PRRSV + SC and SC) and two pigs (Control and PRRSV) were then autopsied at 2, 6, 8, and 16 dpi; blood samples were collected from the jugular vein, and tissue samples were harvested. Samples were aseptically collected during necropsy from organs (brain, lungs, liver, spleen, and four lymph nodes including submandibular, tracheobronchial, gastrohepatic, and mesenteric), blood, and intestinal contents (ileal and rectal) to evaluate bacterial loads. Blood samples were analyzed using an automatic blood cell counter (Hemavet 950, Drew Scientific Inc., Waterbury, CT, USA). Samples of the brain, tonsil, lungs, heart, liver, spleen, kidneys, small and large intestines, and the five lymph nodes (submandibular, tracheobronchial, gastrohepatic, mesenteric, and renal) were obtained for histopathological examination.

### Histopathology

All collected tissues were fixed in 10% neutral buffered formalin for 24 h. The fixed tissues were processed routinely, and 4-µm sections were stained with hematoxylin and eosin. All histopathological changes were classified into four categories according to the previous studies with minor modifications [13–15] as follows: normal (0), mild (1), moderate (2), and severe (3) (Additional file 2).

### *Salmonella* quantification

All samples in experimental studies were stored at 4℃ immediately following collection, and intestinal contents (ileal and rectal) were transferred to the freezer (-80℃) within 1–5 h. We homogenized 1 g of tissue and diluted it 1:10 in PBS. We also diluted 1 mL of heparinized blood similarly. Serial dilutions were plated on Xylose Lysine Deoxycholate agar (Becton Dickinson) supplemented with 10 µg/mL streptomycin sulfate (Sigma-Aldrich, St. Louis, MO, USA) and incubated at 37℃. We enumerated presumptive *Salmonella* colonies daily for 3 days of incubation. At least one representative colony per sample per pig was confirmed as *Salmonella* spp. using the VITEK^®^ MS system (matrix-assisted laser desorption**/**ionization time-of-flight mass spectrometry; MALDI-TOF MS, bioMérieux, Marcy-l’Étoile, Lyon, France).

DNA samples from the intestinal (ileal and rectal) contents (1 g) were extracted using QIAamp DNA stool Mini kit (Qiagen, Hilden, Germany) according to the manufacturer’s instructions. The LiliF *Salmonella* Real-time Polymerase Chain Reaction (PCR) kit (iNtRON Biotechnology Inc.) was used to quantify *Salmonella* Choleraesuis and Typhimurium concentrations in feces. DNA from known concentrations (1.0 × 10^2^, 1.0 × 10^3^, 1.0 × 10^4^, 1.0 × 10^5^, 1.0 × 10^6^, 1.0 × 10^7^, and 1.0 × 10^8^ CFU/mL) of *Salmonella* isolates in the pathogenicity study was used to construct a standard curve. PCR correlation coefficients (*R*^*2*^), slope, and efficiency were calculated.

### Statistical analysis

Statistical analysis was performed using GraphPad Prism (v8.01; GraphPad Software, San Diego, CA, USA). Mean and SD values were calculated. The population normality was assessed using the Shapiro–Wilk test and the population variance was assessed using the Brown-Forsythe test. Based on the results from two tests, non-parametric test was chosen and the Kruskal-Wallis test with Dunn’s post-test was performed to compare the mean values on each dpi. A *P*-value of < 0.05 was considered statistically significant.

## Results

### Experimental design 1

#### Clinical assessment

**Fig. 2:**
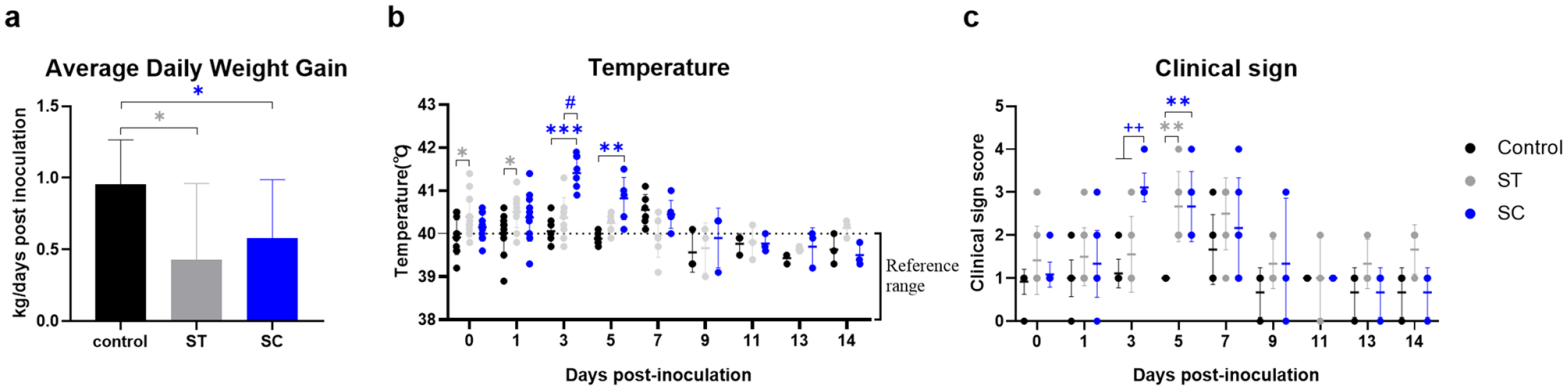
Average daily weight gain, body temperature, and clinical sign scores from experimental study 1. Panels show average daily weight gain (**a**), body temperature (**b**), and clinical sign scores (**c**) in non-infected (control, black) and *Salmonella* (*S*. Typhimurium, ST [gray] and *S*. Choleraesuis, SC [blue])-infected weaned pigs in experimental study 1. Each symbol represents sample results from a single pig; horizontal bars represent the mean ± standard deviation for each group at days post-inoculation. The reference range for body temperature is represented by dotted lines. ^*^ indicates *P* < 0.05, ^**^ indicates *P* < 0.01, or ^***^ indicates *P* < 0.001 versus control group. ^#^ indicates *P* < 0.05 versus ST group. ^++^ indicates *P* < 0.01 versus control and ST group

Figure 2 illustrates the average daily weight gain (ADG), rectal body temperature, and clinical sign scores across all groups. Pigs in the control group exhibited no clinical signs throughout the study period, and their ADG (0.96 kg/dpi) exceeded those of the SC (0.58 kg/dpi) and ST (0.43 kg/dpi) groups. The ADGs of the ST and SC groups were significantly lower than those of the control group (*P* < 0.05; Fig. 2a).

Mean body temperatures in the control group remained below 40℃ for the duration of the study, except at 1 (40.0 °C), 3 (40.0 °C), and 7 (40.6℃) dpi. The ST group exhibited its highest mean body temperature at 1 dpi (mean ± SD: 40.4 ± 0.6℃, *P* = 0.0288), followed by a gradual decline until 13 dpi (Fig. 2b). In the SC group, the mean body temperature increased from 0 to 3 dpi, peaking at 3 dpi (41.5 ± 0.6℃, *P* < 0.001), and then declined progressively until 14 dpi (Fig. 2b). The mean temperatures of the SC group at 3 dpi were significantly higher than those of the ST group (*P* < 0.05; Fig. 2b).

In the SC and ST groups, clinical signs were limited to fever and mild diarrhea. The mean clinical sign scores increased in the SC group until 3 dpi and in the ST group until 5 dpi, followed by a decline in both groups until 11 dpi (Fig. 2c). The SC group recorded the highest mean clinical sign score (3.1) at 3 dpi, which was significantly higher than those of all other groups (*P* < 0.01; Fig. 2c).

#### Gross and histopathology

**Fig. 3:**
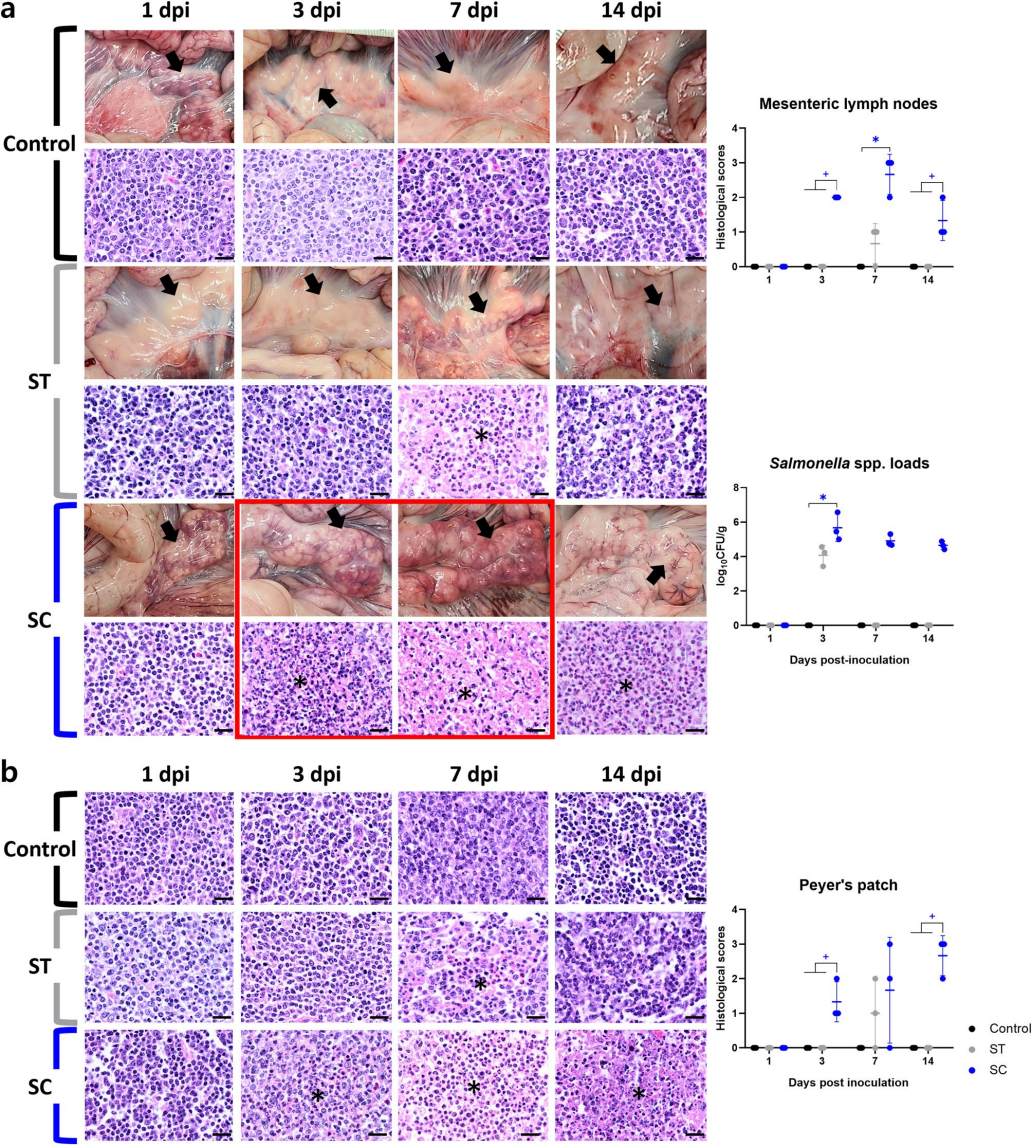
Pathological analysis and *Salmonella* quantification in non-infected and *Salmonella*-infected weaned pigs from experimental study 1. (**a**) Representative gross and histopathological lesions and *S*. Choleraesuis (SC)/ *S*. Typhimurium (ST) loads in mesenteric lymph nodes. Black arrows indicate mesenteric lymph nodes of each pigs at 1, 3, 7, and 14 days post-inoculation (dpi). Red box indicates severe enlargement of mesenteric lymph nodes caused by suppurative lymphadenitis (asterisk). (**b**) Representative histopathological lesions (asterisk, infiltration of numerous neutrophils) and scores of Peyer’s patches. Each symbol represents sample results from a single pig, and horizontal bars represent the mean ± standard deviation for each group at days post-inoculation. ^*^ indicates *P* < 0.05 versus control group. ^+^ indicates *P* < 0.05 versus control and ST group. Hematoxylin and eosin staining, scale bar = 50 μm

No gross and histopathological lesions were found in the control group. Gross lesions were confined to the mesenteric lymph nodes, with all pigs of the SC group between 3 and 7 dpi showed enlargement of mesenteric lymph nodes with redness (Fig. 3a). In the ST group, only one pig demonstrated mild congestion of the mesenteric lymph nodes at 14 dpi.

Histopathological analysis revealed lesions exclusively in the mesenteric lymph nodes and Peyer’s patches of pigs from the SC and ST groups. Histopathological scores for these tissues are presented in Fig. 3. Suppurative lymphadenitis was consistently observed in all SC group pigs from 3 to 14 dpi, whereas only two pigs in the ST group exhibited mild suppurative lymphadenitis at 7 dpi (Fig. 3a). The SC group reached its highest mean histopathological score for mesenteric lymph nodes at 7 dpi, followed by a reduction at 14 dpi. Scores at both 3 and 14 dpi were significantly higher in the SC group than in the ST group (*P* < 0.05; Fig. 3a).

Neutrophilic infiltration in Peyer’s patches was present in all SC group pigs from 3 to 14 dpi, except for one pig at 7 dpi. In contrast, only two pigs in the ST group exhibited suppurative lesions at 7 dpi (Fig. 3b). The SC group demonstrated significantly higher histopathological scores for Peyer’s patches at 3 (*P* < 0.05) and 14 dpi (*P* < 0.05) than those in the ST and control groups. The highest mean score was recorded at 14 dpi (Fig. 3b).

#### *Salmonella* quantification in mesenteric lymph nodes

*S.* Choleraesuis and *S.* Typhimurium were only isolated from mesenteric lymph nodes of pigs from the SC and ST groups, respectively. Quantitative data on *Salmonella* loads in mesenteric lymph nodes are presented in Fig. 3a. All pigs of the SC group between 3 and 14 dpi showed *S.* Choleraesuis loads, and only three pigs of the ST group demonstrated the presence of *S.* Typhimurium at 3 dpi (Fig. 3a). The mean log_10_CFU/g values of *S.* Choleraesuis loads at 3 dpi (*P* = 0.019) were significantly higher than those of the control group, and the mean *S.* Choleraesuis loads tended to decrease from 3 dpi (5.6 log_10_CFU/g) to 14 dpi (4.6 log_10_CFU/g) (Fig. 3a).

### Experimental design 2

#### Clinical assessment and blood counts

**Fig. 4:**
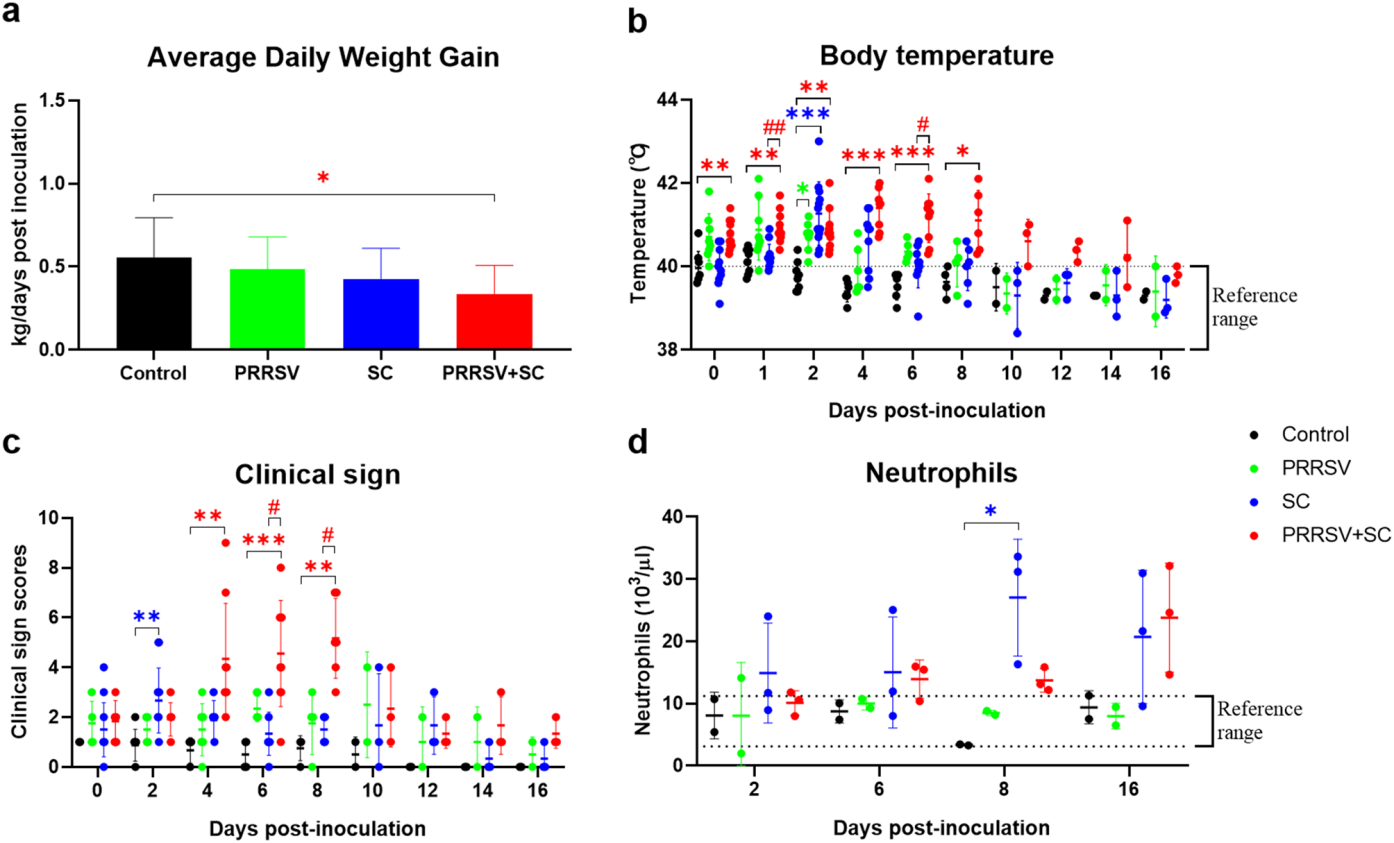
Average daily weight gain, temperature, clinical signs, and neutrophil counts from experimental study 2. Panels show average daily weight gain (**a**), body temperature (**b**), clinical sign scores (**c**), and neutrophil counts (**d**) in non-infected (control, black), PRRSV-infected (green), and *Salmonella-*infected (*S*. Choleraesuis, SC [blue] and PRRSV + SC, SC coinfected with PRRSV [red]) early weaned pigs in experimental study 2. Each symbol represents sample results from a single pig, and horizontal bars represent the mean ± standard deviation for each group at days post-inoculation. The reference ranges of body temperature and neutrophil counts are represented by dotted lines. ^*^ indicates *P* < 0.05, ^**^ indicates *P* < 0.01, or ^***^ indicates *P* < 0.001 versus control group. ^#^ indicates *P* < 0.05 or ^##^ indicates *P* < 0.01 versus SC group. PRRSV, porcine reproductive and respiratory syndrome virus

Figure 4 presents the ADG, rectal body temperature, clinical sign scores, and neutrophil counts across all groups. Pigs in the control group had no clinical signs during the study period, and their ADG (0.5 kg/dpi) was higher than that of all other groups. The PRRSV + SC group demonstrated the lowest ADG (0.3 kg/dpi), which was significantly lower than that of the control group (*P* = 0.0329; Fig. 4a).

Mean body temperatures in the control group remained within the physiological reference range throughout the study, except at 1 dpi. In the SC group, mean body temperatures exceeded 40 °C between 1 and 4 dpi (Fig. 4b). In PRRSV-infected pigs (PRRSV and PRRSV + SC groups), mean body temperatures in the PRRSV group exceeded 40 °C from 0 to 8 dpi, except at 4 dpi. In the PRRSV + SC group, elevated temperatures (> 40 °C) persisted from 0 to 14 dpi (Fig. 4b). The highest mean body temperatures were recorded at 2 dpi in the SC group (41.3 ± 0.7 °C, *P* < 0.001) and at 4 dpi in the PRRSV + SC group (41.4 ± 0.4 °C, *P* = 0.0002). Between 0 and 8 dpi, mean body temperatures in the PRRSV + SC group—excluding 2 dpi—were significantly higher than those in the SC group (Fig. 4b).

In the SC group, pigs exhibited mild diarrhea and fever, with the highest clinical sign score observed at 2 dpi (*P* = 0.0011; Fig. 4c). In the PRRSV + SC group, pigs began showing mild respiratory signs and fever at 4 dpi, followed by moderate dyspnea, mild diarrhea, and cyanosis (ears, nose tip, and rump) between 6 and 8 dpi (Fig. 4c). After 12 dpi, only mild fever persisted in this group. Clinical sign scores in the PRRSV + SC group between 6 and 8 dpi were significantly higher than those in the SC group, and scores declined progressively after 8 dpi until 12 dpi (Fig. 4c). In the PRRSV group, pigs exhibited mild respiratory signs and fever from 0 to 10 dpi, with no clinical signs observed beyond 12 dpi.

Red blood cell, platelet, and total white blood cell counts remained within reference ranges across all groups, except for neutrophils. Mean neutrophil counts in the SC group exceeded the reference range throughout the study, with a significantly elevated count at 8 dpi compared with that in the control group (*P* = 0.0399; Fig. 4d). In the PRRSV + SC group, mean neutrophil counts remained above the reference range from 6 to 16 dpi and were higher than those in the SC group at 16 dpi (Fig. 4d). Mean neutrophil counts in the PRRSV and control groups remained within normal limits (Fig. 4d).

#### Gross and histopathology

**Fig. 5:**
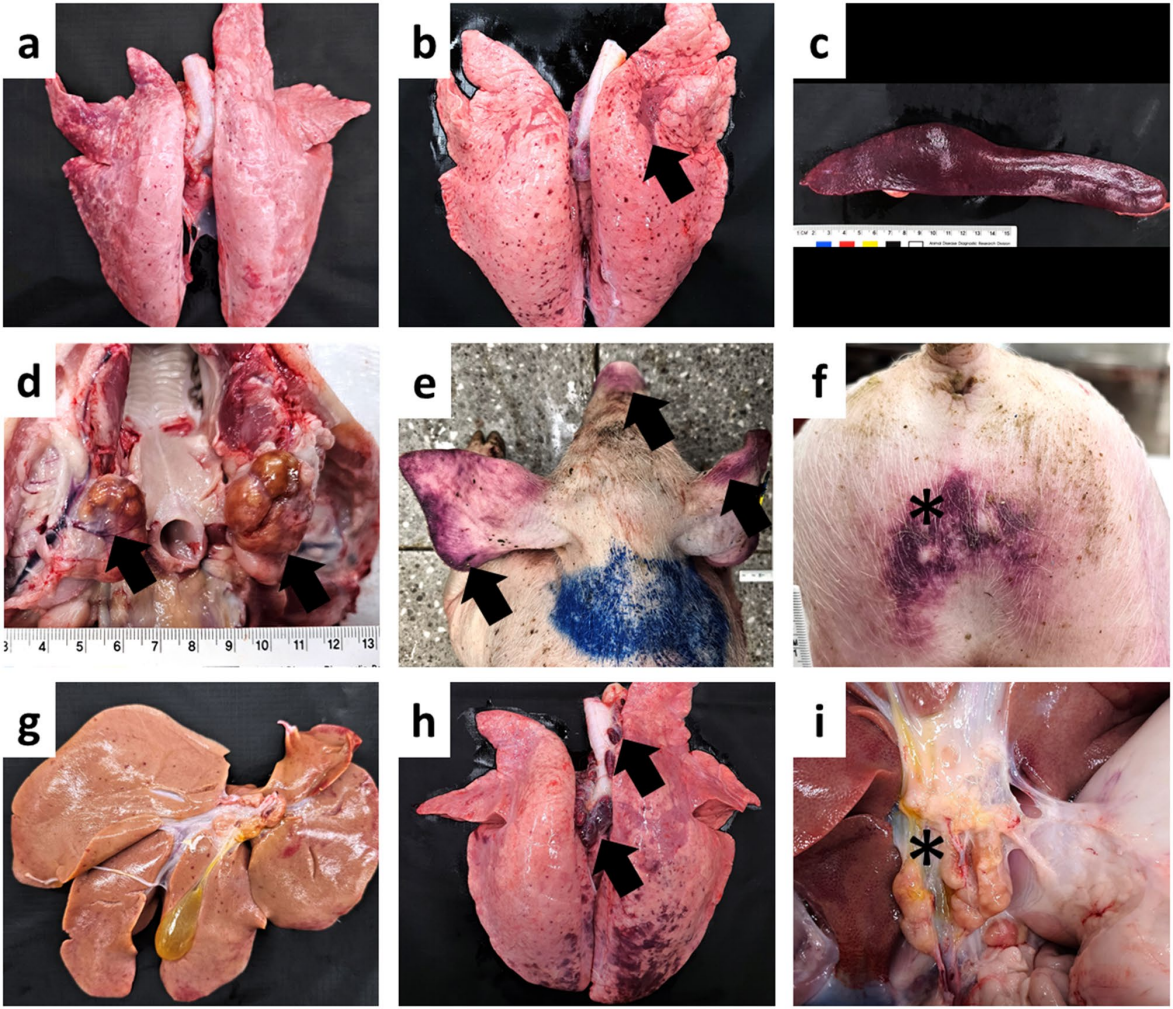
Gross lesions of early-weaned pigs in experimental study 2. (**a**) PRRSV group at 6 dpi, non-collapsed lungs. (**b**) SC group at 6 dpi, multifocal petechiae across entire lung surface with mild consolidation (black arrow). (**c**) SC group at 8 dpi, splenomegaly with dark discoloration. (**d**) PRRSV + SC group at 6 dpi, moderate enlargement of submandibular lymph nodes (black arrows). (**e**) PRRSV + SC group at 6 dpi, severe cyanosis (black arrows) in both ears and the nose tip. (**f**) PRRSV + SC group at 6 dpi, severe ecchymosis (asterisk) in the rump. (**g**) PRRSV + SC group at 6 dpi, multifocal petechiae in liver. (**h**) PRRSV + SC group at 16 dpi, severe hemorrhagic enlargement of tracheobronchial lymph nodes (black arrows) and severe ecchymosis in caudal lobes. (**i**) PRRSV + SC group at 16 dpi, enlargement of gastrohepatic lymph nodes. PRRSV, porcine reproductive and respiratory syndrome virus; SC, *Salmonella* Choleraesuis; dpi, days post-inoculation

No gross and histopathological lesions were observed in the control group. In pigs infected with PRRSV or *S.* Choleraesuis, no abnormalities were detected in the brain, tonsil, heart, kidneys, renal lymph nodes, or small/large intestines. The proportion of pigs exhibiting gross lesions in each organ across the infected groups is summarized in Table 1. Representative gross lesions from pigs in experimental study 2 are shown in Fig. 5. Pigs in the PRRSV group demonstrated slight pulmonary retraction accompanied by a small number of petechiae (Fig. 5a). In the SC group, one pig at 6 dpi exhibited multifocal pulmonary petechiae with mild consolidation (Fig. 5b), whereas another pig presented with splenomegaly and dark discoloration (Fig. 5c). In the PRRSV + SC group, enlargement of lymph nodes—including submandibular, tracheobronchial, gastrohepatic, and mesenteric nodes—was observed by 6 dpi (Fig. 5d) and cyanotic discoloration of the ears, nose tip, and rump was noted in one pig at 6 dpi (Fig. 5e and f). At 8 dpi, two pigs exhibited hepatic petechiae and mild splenomegaly with dark discoloration (Fig. 5g). By 16 dpi, two pigs demonstrated cranioventral pulmonary consolidation with hemorrhagic enlargement of tracheobronchial lymph nodes (Fig. 5h) and concurrently enlargement of the gastrohepatic lymph node (Fig. 5i).

**Table 1:** Proportions of pigs with gross lesions across three inoculated groups

Gross lesions		Groups^*^	Percentages (%) of pigs with identified lesions			
			2 dpi^**^	6 dpi	8 dpi	16 dpi
Cyanosis in ears						
		PRRSV	0	0	0	0
		SC	0	0	0	0
		PRRSV + SC	0	33.3 (1/3)	66.7 (2/3)	33.3 (1/3)
Mild pulmonary retraction with scattered petechiae						
		PRRSV	50 (1/2)	100 (2/2)	50 (1/2)	0
		SC	66.7 (2/3)	100 (3/3)	100 (3/3)	33.3 (1/3)
		PRRSV + SC	66.7 (2/3)	100 (3/3)	66.7 (2/3)	100 (3/3)
Petechiae on liver						
		PRRSV	0	0	0	0
		SC	0	33.3 (1/3)	33.3 (1/3)	0
		PRRSV + SC	0	33.3 (1/3)	66.7 (2/3)	0
Splenomegaly with blue discoloration						
		PRRSV	0	0	0	0
		SC	0	33.3 (1/3)	33.3 (1/3)	0
		PRRSV + SC	0	33.3 (1/3)	66.7 (2/3)	0
Enlargements of submandibular Ln^***^						
		PRRSV	0	100 (2/2)	100 (2/2)	50 (1/2)
		SC	100 (3/3)	0	66.7 (2/3)	0
		PRRSV + SC	0	100 (3/3)	66.7 (2/3)	66.7 (2/3)
Enlargements of tracheobronchial Ln						
		PRRSV	50 (1/2)	50 (1/2)	100 (2/2)	50 (1/2)
		SC	100 (3/3)	0	66.7 (2/3)	0
		PRRSV + SC	66.7 (2/3)	66.7 (2/3)	66.7 (2/3)	66.7 (2/3)
Enlargements of gastrohepatic Ln						
		PRRSV	0	0	0	0
		SC	33.3 (1/3)	33.3 (1/3)	100 (3/3)	0
		PRRSV + SC	0	33.3 (1/3)	66.7 (2/3)	66.7 (2/3)
Enlargements of mesenteric Ln						
		PRRSV	0	0	0	0
		SC	100 (3/3)	33.3 (1/3)	100 (3/3)	0
		PRRSV + SC	0	100 (3/3)	100 (3/3)	100 (3/3)

^*^ PRRSV, pigs inoculated with porcine reproductive and respiratory syndrome virus (PRRSV); SC, pigs inoculated with *Salmonella* Choleraesuis (SC); PRRSV + SC, pigs coinfected with PRRSV and SC

^**^ dpi, days post-inoculation

^***^ Ln, Lymph node

**Fig. 6:**
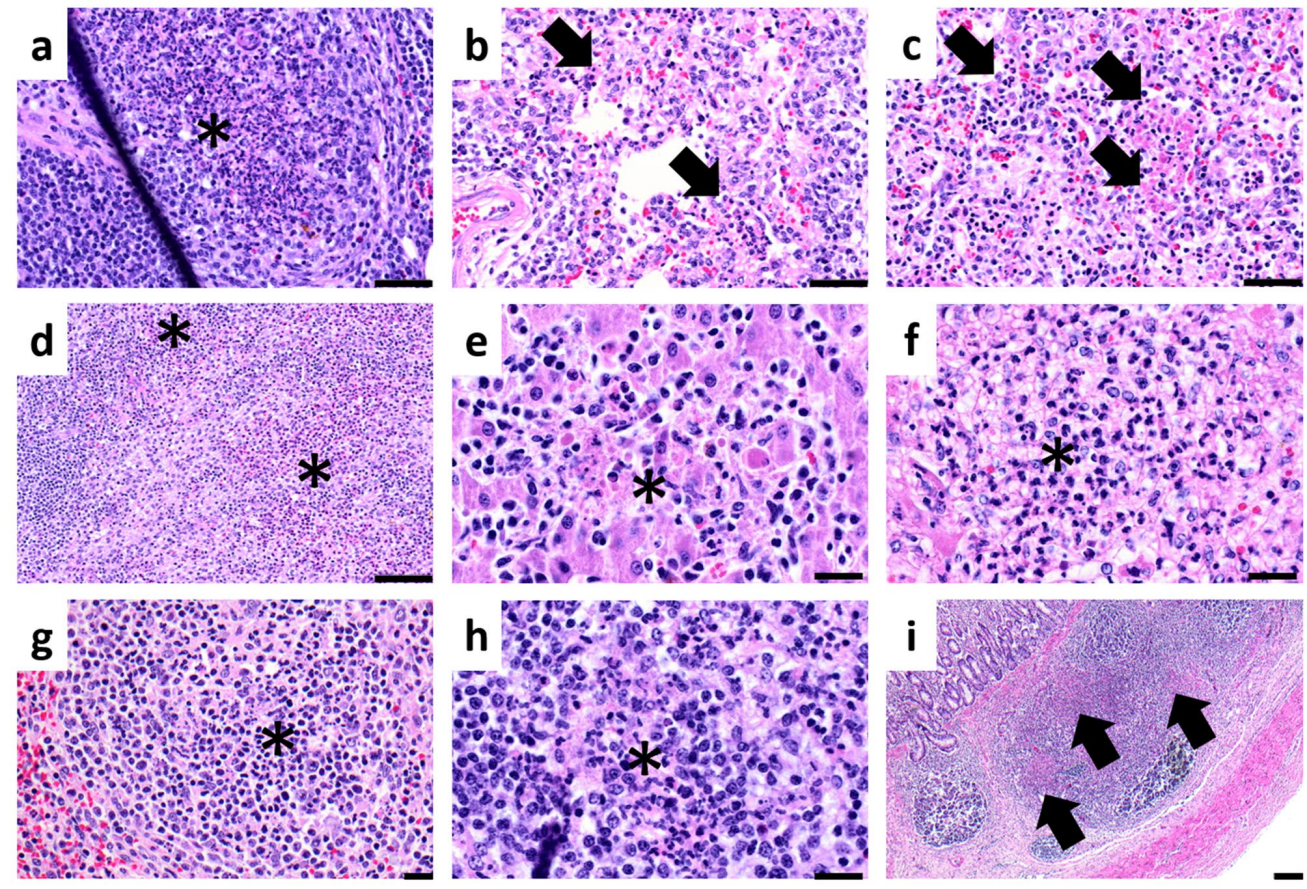
Histopathological lesions of early weaning pigs in experimental study 2. (**a**) SC group at 2 dpi, mild suppurative lymphadenitis (asterisk) in submandibular lymph nodes. Hematoxylin and eosin (H&E) stain, scale bar = 50 μm. (**b**) PRRSV group at 2 dpi, mild infiltrations (black arrows) of mononuclear cells and neutrophils within the alveolus. H&E stain, scale bar = 50 μm. (**c**) PRRSV + SC group at 8 dpi, severe infiltration (black arrows) of mononuclear and necrotized cells within the alveolus. H&E stain, scale bar = 50 μm. (**d**) PRRSV + SC group at 8 dpi, moderate suppurative lymphadenitis (asterisks) in tracheobronchial lymph nodes. H&E stain, scale bar = 100 μm. (**e**) SC group at 16 dpi, mild foci of necrotized hepatocytes infiltrated with mononuclear cells (asterisk). H&E stain, scale bar = 20 μm. (**f**) PRRSV + SC group at 8 dpi, severe hepatocellular degeneration (asterisk) with necrotized hepatocytes infiltrated with mononuclear cells and neutrophils. H&E stain, scale bar = 20 μm. (**g**) SC group at 8 dpi, moderate infiltration (asterisk) of neutrophils mixed with necrotized lymphoid cells within the white pulp. H&E stain, scale bar = 20 μm. (**h**) PRRSV + SC group at 8 dpi, mild suppurative lymphadenitis (asterisk) in gastrohepatic lymph nodes. H&E stain, scale bar = 20 μm. (**i**) SC group at 8 dpi, severe and diffuse infiltration of neutrophils in Peyer’s patch. H&E stain, scale bar = 200 μm. SC, *Salmonella* Choleraesuis; PRRSV, porcine reproductive and respiratory syndrome virus; dpi, days post-inoculation

**Fig. 7:**
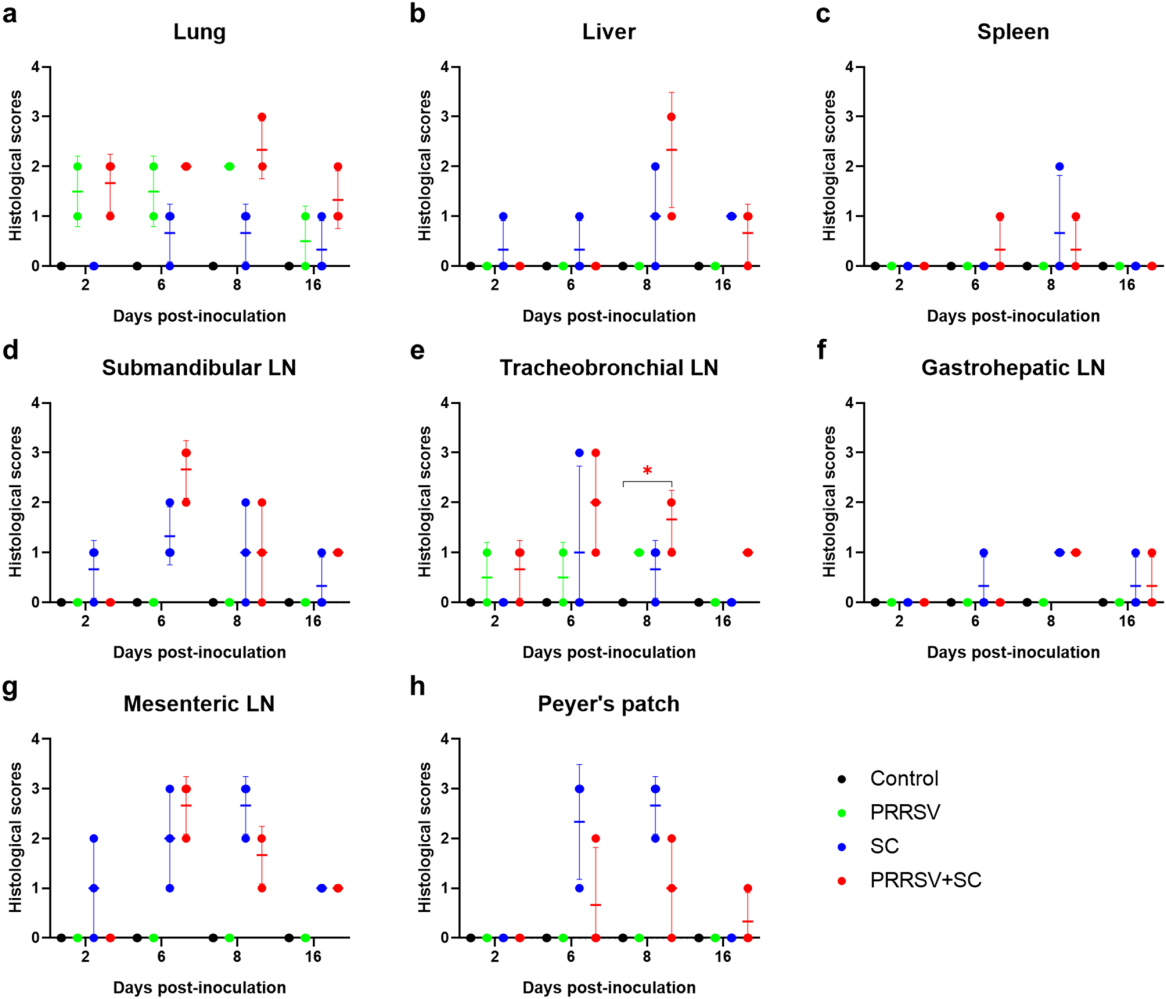
Histopathological scores in organs and lymph nodes of infected early weaned pigs from Study 2. Histopathological scoring of early weaned pigs infected with PRRSV (green), *Salmonella* Choleraesuis (SC, blue), and *S*. Choleraesuis coinfected with PRRSV (PRRSV + SC, red) in the lung (**a**), liver (**b**), spleen (**c**), submandibular LN (**d**), tracheobronchial LN (**e**), gastrohepatic LN (**f**), mesenteric LN (**g**), and Peyer’s patches (**h**). Each symbol represents histopathological scores from a single pig, and horizontal bars represent the mean ± standard deviation for each group at days post-inoculation. PRRSV, porcine reproductive and respiratory syndrome virus; LN, lymph nodes

Representative histopathological lesions and scores are shown in Figs. 6 and 7. In the SC and PRRSV + SC groups, lesions were observed in the lungs (interstitial pneumonia), liver (hepatocellular necrosis with neutrophilic and macrophagic infiltration), spleen (suppurative splenitis), lymph nodes (suppurative lymphadenitis affecting submandibular, tracheobronchial, gastrohepatic, and mesenteric nodes), and Peyer’s patches (lymphocytic necrosis with neutrophilic infiltration). In the PRRSV group, lesions were limited to the submandibular and tracheobronchial lymph nodes (suppurative lymphadenitis) and lungs (interstitial pneumonia). The proportion of pigs with histopathological lesions from each tissue across all infected groups is depicted in Table 2.

**Table 2:** Proportions of pigs with histopathological lesions across three inoculated groups

Histopathological lesions		Groups^*^	Percentages (%) of pigs with lesions identified			
			2 dpi^**^	6 dpi	8 dpi	16 dpi
Interstitial pneumonia						
		PRRSV	100 (2/2)	100 (2/2)	100 (2/2)	50 (1/2)
		SC	0	66.7 (2/3)	66.7 (2/3)	33.3 (1/3)
		PRRSV + SC	100 (3/3)	100 (3/3)	100 (3/3)	100 (3/3)
Paratyphoid nodule						
		PRRSV	0	0	0	0
		SC	33.3 (1/3)	100 (3/3)	66.7 (2/3)	100 (3/3)
		PRRSV + SC	0	100 (3/3)	100 (3/3)	66.7 (2/3)
Suppurative splenitis						
		PRRSV	0	0	0	0
		SC	0	0	33.3 (1/3)	0
		PRRSV + SC	0	33.3 (1/3)	33.3 (1/3)	0
Infiltrations of neutrophils in Peyer’s patch						
		PRRSV	0	0	0	0
		SC	0	100 (3/3)	100 (3/3)	0
		PRRSV + SC	0	33.3 (1/3)	66.7 (2/3)	33.3 (1/3)
Suppurative lymphadenitis in submandibular Ln^***^						
		PRRSV	0	0	0	0
		SC	66.7 (2/3)	100 (3/3)	66.7 (2/3)	33.3 (1/3)
		PRRSV + SC	0	100 (3/3)	66.7 (2/3)	100 (3/3)
Suppurative lymphadenitis in tracheobronchial Ln						
		PRRSV	50 (1/2)	50 (1/2)	100 (2/2)	0
		SC	0	33.3 (1/3)	66.7 (2/3)	0
		PRRSV + SC	66.7 (2/3)	100 (3/3)	100 (3/3)	100 (3/3)
Suppurative lymphadenitis in gastrohepatic Ln						
		PRRSV	0	0	0	0
		SC	0	33.3 (1/3)	100 (3/3)	33.3 (1/3)
		PRRSV + SC	0	0	100 (3/3)	33.3 (1/3)
Suppurative lymphadenitis in mesenteric Ln						
		PRRSV	0	0	0	0
		SC	66.7 (2/3)	100 (3/3)	100 (3/3)	100 (3/3)
		PRRSV + SC	33.3 (1/3)	100 (3/3)	100 (3/3)	100 (3/3)

^*^ PRRSV, pigs inoculated with porcine reproductive and respiratory syndrome virus (PRRSV); SC, pigs inoculated with *Salmonella* Choleraesuis (SC); PRRSV + SC, pigs coinfected with PRRSV and SC

^**^ dpi, days post-inoculation

^***^ Ln, Lymph node

In the SC and PRRSV + SC groups, mean histopathological scores for submandibular and tracheobronchial lymph nodes peaked at 6 dpi. Mean scores for the lungs, liver, spleen, gastrohepatic lymph nodes, and Peyer’s patches reached their highest values at 8 dpi (Fig. 7). Throughout the study, the PRRSV + SC group exhibited higher pulmonary histopathological scores than those of other groups. Mesenteric lymph node scores peaked at 8 dpi in the SC group and at 6 dpi in the PRRSV + SC group. In the PRRSV group, mean histopathological scores for the lungs and tracheobronchial lymph nodes peaked at 8 dpi and declined by 16 dpi (Fig. 7a and e).

### *S. Choleraesuis quantification and detection*

**Fig. 8:**
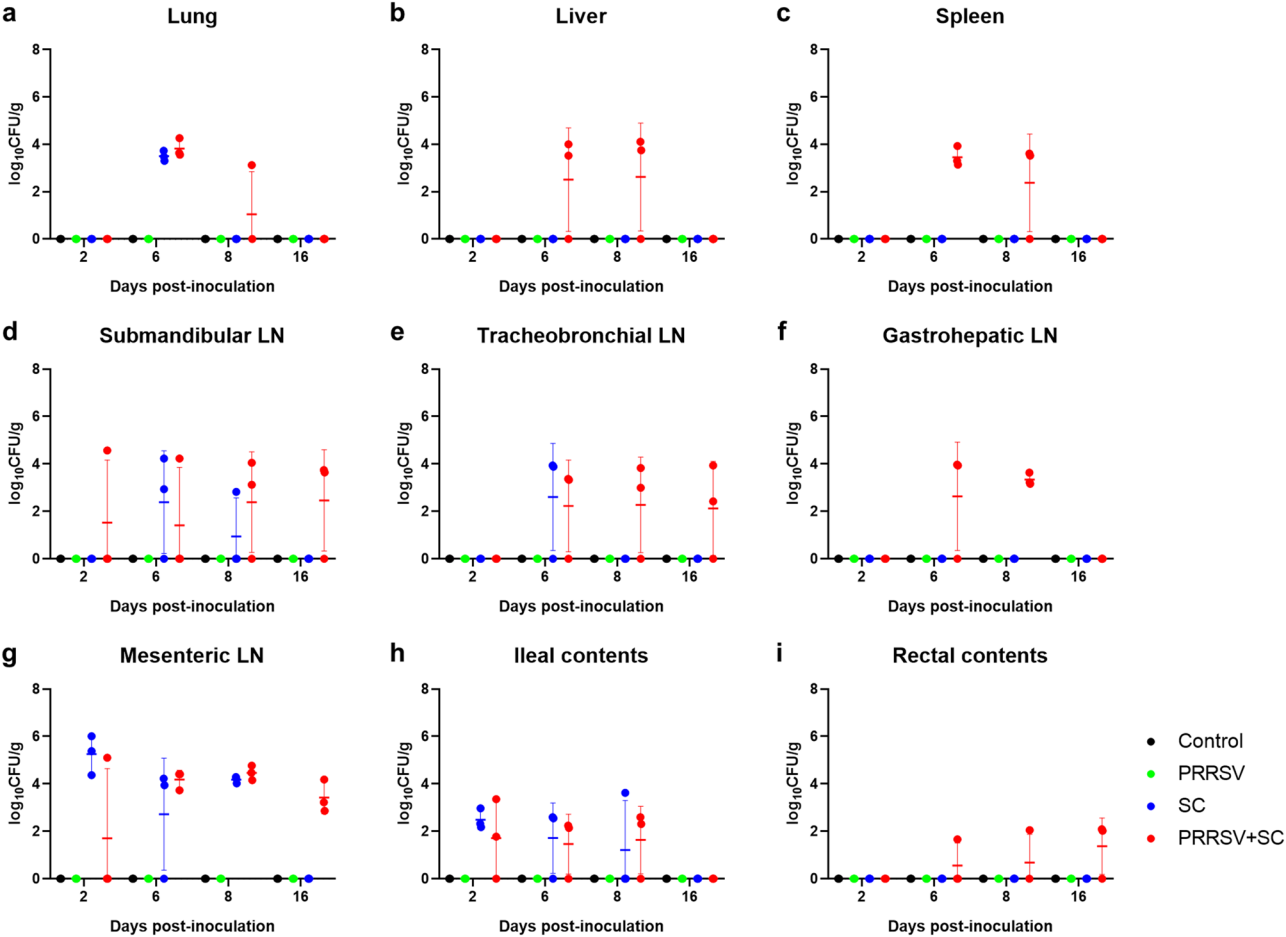
*Salmonella* loads in tissues and contents of infected early weaned pigs from experimental study 2. Quantification of *Salmonella* Choleraesuis (SC) loads (log_10_CFU/g) in early weaned pigs infected with PRRSV (green), *S*. Choleraesuis (blue), and *S*. Choleraesuis coinfected with PRRSV (PRRSV + SC, red) across the lungs (**a**), liver (**b**), spleen (**c**), submandibular LN (**d**), tracheobronchial LN (**e**), gastrohepatic LN (**f**), mesenteric LN (**g**), ileal contents (**h**), and rectal contents (**i**). Each symbol represents SC loads from a single pig, and horizontal bars represent the mean ± standard deviation for each group at days post-inoculation. PRRSV, porcine reproductive and respiratory syndrome virus; LN, lymph nodes

All samples from pigs in the control and PRRSV groups tested negative for *S*. Choleraesuis throughout the study. No *S*. Choleraesuis was isolated from blood or brain samples in the SC and PRRSV + SC groups. Quantitative data on *S*. Choleraesuis loads (log_10_CFU/g) in the lungs, liver, spleen, lymph nodes (submandibular, tracheobronchial, gastrohepatic, and mesenteric), and intestinal contents (ileal and rectal) at each dpi are presented in Fig. 8.

In the SC group, *S.* Choleraesuis was isolated from the lungs, lymph nodes (submandibular, tracheobronchial, and mesenteric), and ileal contents from 2 to 8 dpi. The number of tissues (submandibular and tracheobronchial lymph nodes and lung) positive for *S*. Choleraesuis increased from 2 to 6 dpi (Fig. 8). Although the mean *S.* Choleraesuis loads from mesenteric lymph nodes at 8 dpi were slightly higher than those at 6 dpi, the number of tissues and ileal contents positive for *S*. Choleraesuis declined between 6 and 8 dpi (Fig. 8). *S*. Choleraesuis was not detected in the tissues and intestinal contents at 16 dpi in the SC group.

Contrary to the SC group, the PRRSV + SC group exhibited a greater number of *S.* Choleraesuis-positive tissues and intestinal contents throughout the study. S. Choleraesuis was additionally isolated from the liver, spleen, gastrohepatic lymph nodes, and rectal contents—sites not positive in the SC group. By 8 dpi, mean bacterial loads increased in all tissues and intestinal contents except the lungs and spleen. At 16 dpi, unlike the SC group, lymph nodes (mesenteric, submandibular, and tracheobronchial) and rectal contents remained positive for *S.* Choleraesuis.

## Discussion

Previous pathogenicity studies involving *Salmonella* spp. in pigs have predominantly focused on enterocolitis, with limited experimental investigations into septicemic salmonellosis [3, 16]. Although routine diagnosis of salmonellosis typically relies on fecal or intestinal content sampling, the detection of *Salmonella* spp. in pigs can be delayed and complicated due to prolonged incubation times on selective media (e.g., Rappaport-Vassiliadis broth and CHROMagar) and competition from commensal intestinal bacteria [17]. Prior research has identified mesenteric lymph nodes as key sites for *Salmonella* persistence, attributed to the intracellular survival of isolates within macrophages and fibroblasts [3]. For isolating *S.* Choleraesuis in septicemic salmonellosis cases, samples from the lungs, liver, and spleen have been recommended for pure cultures [2]. Given the sustained colonization of *S.* Choleraesuis in mesenteric lymph nodes observed in this study, these nodes should be considered critical targets for bacterial isolation in suspected cases of septicemic salmonellosis in weaned pigs.

A previous experimental study using *S.* Choleraesuis and *S.* Typhimurium in 4-week-old specific pathogen-free (SPF) pigs reported systemic lesions, including interstitial pneumonia, multifocal hepatic necrosis, and suppurative lymphadenitis, in pigs infected with *S.* Choleraesuis and enterocolitis with diarrhea in pigs inoculated with *S.* Typhimurium [18]. Although pigs in the SC and ST groups in this study exhibited limited colonization restricted to mesenteric lymph nodes and Peyer’s patches, these findings may reflect differences in host type (SPF vs. commercial pigs) and inoculation dose (1 × 10^10^ CFU/mL and 2 × 10^8^ CFU/mL) [18]. However, the SC group exhibited greater virulence than did the ST group, as evidenced by elevated mean body temperature and clinical sign scores, higher bacterial loads in mesenteric lymph nodes, and more severe histopathological lesions in mesenteric lymph nodes and Peyer’s patches.

When comparing inoculation age of *S.* Choleraesuis in weaned pigs, no meaningful differences in body temperatures or clinical sign scores were observed between 6-week-old (experimental study 2) and 8-week-old pigs (experimental study 1). However, the ADG in 6-week-old weaned pigs until 14 dpi (0.42 kg/dpi) was lower than that in 8-week-old pigs (0.58 kg/dpi), and pulmonary colonization by *S.* Choleraesuis occurred exclusively in 6-week-old weaned pigs. Previous studies have shown that younger animals are highly susceptible to *Salmonella* infections due to immature intestinal flora and underdeveloped immune systems [19]. Although the age difference between these groups was only 2 weeks, the timing of infection may influence the pathogenicity of *S.* Choleraesuis isolates in weaned pigs.

In experimental study 2, all groups except PRRSV + SC appeared to resolve infection before 16 dpi, based on body temperature trends, clinical sign scores, and bacterial load data. The absence of *S.* Choleraesuis isolation from SC group tissues at 16 dpi contrasts with findings from a previous study [20]. All 5-week-old pigs intranasally inoculated with *S.* Choleraesuis exhibited bacterial loads in ileocolic lymph nodes at 21 dpi, and 14% of infected pigs had detectable bacteria in non-intestinal tissues, including the liver, tonsil, and bronchial lymph nodes [20]. Although oral inoculation is considered a relevant route for *Salmonella* transmission, intranasal inoculation may elicit a more robust infection [21]. These discrepancies likely reflect differences in inoculation routes between studies.

In the PRRSV + SC group, all pigs at 16 dpi revealed bacterial loads in mesenteric lymph nodes, indicating prolonged infection and confirming these nodes as reservoirs for *S.* Choleraesuis persistence. A previous experimental study of coinfection with *S.* Choleraesuis and PRRSV has shown that PRRSV enhances susceptibility to *S.* Choleraesuis, resulting in more severe systemic and respiratory diseases in pigs [20]. Despite differences in inoculation routes between the present (oral) and previous (intranasal) studies, similar systemic disease manifestations were observed, including persistent fever, cyanosis, widespread bacterial dissemination, and extensive histopathological lesions. PRRSV replication within macrophages impairs immune function by damaging pulmonary intravascular macrophages and reducing bacterial clearance from blood [20, 22]. The pronounced septicemic signs and lesions in the PRRSV + SC group likely reflect the immunosuppressive effects of PRRSV in pigs.

According to data from the European Union Reference Laboratory and Korean experimental studies involving highly virulent ASFV strains, ASFV-infected pigs exhibit hemorrhagic enlargement of gastrohepatic and renal lymph nodes resembling blood clots [6, 9, 23]. In contrast, pigs infected with *S.* Choleraesuis have been reported to show enlarged and congested gastrohepatic lymph nodes without hemorrhage [2]. In this study, enlargement of gastrohepatic lymph nodes without hemorrhagic changes was observed exclusively in *S.* Choleraesuis-infected pigs, and no pathological lesions were noted in renal lymph nodes. Although ASF diagnosis is confirmed via PCR, veterinarians performing necropsies on suspected ASF cases should assess gastrohepatic and renal lymph nodes for early lesion detection, aiding in movement restriction and differential diagnosis between ASF and septicemic salmonellosis [7].

One limitation of this study is the exclusion of bacterial load analysis in renal lymph nodes and tonsils. To our knowledge, no studies have specifically examined *S.* Choleraesuis distribution in renal lymph nodes [18, 20, 22]. One study evaluating kidney colonization found that none of the 14 pigs orally inoculated with *S.* Choleraesui*s* or *S.* Typhimurium exhibited bacterial loads in the kidneys until 5 dpi [18]. This suggests that septicemic *Salmonella* spp. are less likely to colonize the kidneys and renal lymph nodes. Lymphoid tissues, including tonsils and ileocolic lymph nodes, are considered potential reservoirs for *Salmonella* spp., with *S.* Choleraesui*s* isolations found in the tonsils and ileocolic lymph nodes until 9 weeks post-infection in a previous study [24]. However, no pathological lesions were observed in the tonsils of *S.* Choleraesuis-inoculated pigs in this study, which focused on bacterial distribution in parenchymal organs and lymphoid tissues, except for tonsils following oral inoculation.

## Conclusions

Despite the inherent challenges of oral inoculation, this study provides critical insights into the systemic dissemination and pathogenicity of *S.* Choleraesuis isolates in pigs [20]. These findings enhance understanding of the clinicopathological characteristics of *S.* Choleraesuis infection. To the best of our knowledge, this is the first report confirming *S.* Choleraesuis colonization in gastrohepatic lymph nodes. Given the diagnostic importance of hemorrhagic enlargement in gastrohepatic and renal lymph nodes during acute ASFV infection, the findings from this study offer valuable differentiation between ASF and septicemic salmonellosis. This information may assist veterinarians and farmers in ASF-endemic regions in early lesion recognition and disease management. Future study is necessary to determine the virulence associated genes of *S.* Choleraesuis isolates in order to understand the pathogenicity and tissue colonization of those isolates.

## Supplementary Information

Below is the link to the electronic supplementary material.

Supplementary Material 1: File name: Additional file 1. File format: Additional_File_1_v4.docx. Title of data: Clinical signs used to calculate clinical sign scores of *Salmonella* spp. Infections. Description of data: Clinical sign criteria for determining clinical sign scores of *Salmonella*-infected weaned pigs

Supplementary Material 2: File name: Additional file 2. File format: Additional_File_2_v2.docx. Title of data: Histopathological evaluation criteria of *Salmonella* spp. infections. Description of data: Histopathological evaluation criteria for determining histopathological scores of *Salmonella*-infected weaned pigs

## Data Availability

No datasets were generated or analysed during the current study.

## Abbreviations

ADG: Average daily weight gain
ASF: African swine fever
ASFV: African swine fever virus
CFU: Colony forming unit
dpi: days post-inoculation
OD600: Optical density at 600 nm
PBS: Phosphate buffered saline
PCR: Polymerase chain reaction
PRRSV: Porcine reproductive and respiratory syndrome virus
SPF: Specific pathogen free
TCID_50_: Tissue culture infective dose 50%
