# Supplementary material for: Experimental evaluation of Salmonella Choleraesuis pathogenicity and porcine reproductive and respiratory syndrome virus synergy in weaned pigs
Source: Porcine Health Manag. 2026 Mar 11;12:22. doi: 10.1186/s40813-026-00502-8 (PMC13088407; doi:10.1186/s40813-026-00502-8)
Supplement: Supplementary file 1 — Supplementary Material 1: File name: Additional file 1. File format: Additional_File_1_v4.docx. Title of data: Clinical signs used to calculate clinical sign scores of Salmonella spp. Infections. Description of data: Clinical sign criteria for determining clinical sign scores of Salmonella-infected weaned pigs [file 40813_2026_502_MOESM1_ESM.docx]

**Additional File 1**. Clinical signs used to calculate clinical sign scores of *Salmonella* spp. infections

| Clinical signs | Severity (0: normal, 1: mild, 2: moderate, 3: severe) | Assigned scores |
| --- | --- | --- |
| Fever | <39.5℃ | 0 |
|  | 39.5–40.5 ℃ | 1 |
|  | 40.6–41.0 ℃ | 2 |
|  | >41.0 ℃ | 3 |
| Behavior | Normal | 0 |
|  | Decreased activity, mild-to-moderate clumsiness | 1 |
|  | Decreased response to external stimuli | 2 |
|  | Markedly decreased or lack of response to external stimuli, immobile, prostration | 3 |
| Skin | Normal | 0 |
|  | Body cyanotic areas (<10%); minimal multifocal cutaneous necrosis and/or hemorrhage | 1 |
|  | Body cyanotic areas (11–25%); mild-to-moderate multifocal cutaneous necrosis and/or hemorrhage | 2 |
|  | Body cyanotic areas (>25%); moderate-to-marked multifocal cutaneous necrosis and/or hemorrhage | 3 |
| Feces | Normal (firm or mild moist feces) | 0 |
|  | Feces around the anus (increasingly moist feces without clumping) | 1 |
|  | Feces covering posterior gluteus (moderate diarrhea flattening out on floor) | 2 |
|  | Feces covering posterior gluteus with extensive mucus or blood (severe diarrhea) | 3 |
| Respiratory system | Normal | 0 |
|  | Mild dyspnea (labored respiration) | 1 |
|  | Moderate dyspnea or cough | 2 |
|  | Marked dyspnea or severe labored respiration | 3 |
| Body condition | Normal, full stomach | 0 |
|  | Empty stomach, sunken flanks | 1 |
|  | Empty stomach, sunken flanks, loss of muscle mass | 2 |
|  | Emaciated | 3 |

This list was generated according to a previous study by Lee et al. (2021) and Naberhaus et al., (2020), with minor modifications.

Lee HS, Bui VN, Dao DT, Bui NA, Le TD, Kieu MA, et al. Pathogenicity of an African swine fever virus strain isolated in Vietnam and alternative diagnostic specimens for early detection of viral infection. Porcine Health Manag. 2021;7:36.

Naberhaus SA, Krull AC, Arruda BL, Arruda P, Sahin O, Schwartz KJ, et al. Pathogenicity and competitive fitness of *Salmonella* enterica Serovar 4,[5],12:i:-Compared to *Salmonella* Typhimurium and *Salmonella* Derby in Swine. Front Vet Sci. 2020;6:502.
