# Supplementary material for: Experimental evaluation of Salmonella Choleraesuis pathogenicity and porcine reproductive and respiratory syndrome virus synergy in weaned pigs
Source: Porcine Health Manag. 2026 Mar 11;12:22. doi: 10.1186/s40813-026-00502-8 (PMC13088407; doi:10.1186/s40813-026-00502-8)
Supplement: Supplementary file 2 — Supplementary Material 2: File name: Additional file 2. File format: Additional_File_2_v2.docx. Title of data: Histopathological evaluation criteria of Salmonella spp. infections. Description of data: Histopathological evaluation criteria for determining histopathological scores of Salmonella-infected weaned pigs [file 40813_2026_502_MOESM2_ESM.docx]

**Additional File 2.** Histopathological evaluation criteria of *Salmonella* spp. infections

| Tissues | Severity (0: normal, 1: mild, 2: moderate, 3: severe) | Assigned scores |
| --- | --- | --- |
| Lymphoid tissues | No inflammation and lymphoid depletion | 0 |
|  | Mild infiltration of neutrophils and inflammatory-cell infiltration replacing normal parenchymal tissue | 1 |
|  | Moderate infiltration of neutrophils and inflammatory-cell infiltration replacing normal parenchymal tissue | 2 |
|  | Severe and diffuse infiltration of neutrophils and inflammatory-cell infiltration replacing normal parenchymal tissue, fibrin, and/or tissue necrosis | 3 |
| Lung | No inflammation | 0 |
|  | Mild infiltration of mononuclear cells, macrophages, and intra-alveolar or bronchiolar exudate | 1 |
|  | Moderate infiltration of mononuclear cells, macrophages, neutrophils, and intra-alveolar or bronchiolar exudate | 2 |
|  | Severe and diffuse infiltration of mononuclear cells, macrophages, neutrophils, and necrotized cells within the bronchiole or alveolus | 3 |
| Liver | No inflammation and hepatocellular degeneration | 0 |
|  | Mild hepatocellular degeneration with necrotized hepatocytes and infiltration of mononuclear cells and neutrophils | 1 |
|  | Moderate hepatocellular degeneration with necrotized hepatocytes and infiltration of mononuclear cells and neutrophils | 2 |
|  | Severe and diffuse hepatocellular degeneration with necrotized hepatocytes and infiltration of mononuclear cells and neutrophils | 3 |

The criteria were established according to the following previous studies with minor modifications:

- Harrison OL, Gebhardt JT, Paulk CB, Plattner BL, Woodworth JC, Rensing S, et al. Inoculation of weaned pigs by feed, water, and airborne transmission of Salmonella enterica serotype 4,[5],12: i:. J Food Prot. 2022;85:693-700.
- Woolley LK, Fell S, Gonsalves JR, Walker MJ, Djordjevic SP, Jenkins C, et al. Evaluation of clinical, histological and immunological changes and qPCR detection of Mycoplasma hyopneumoniae in tissues during the early stages of mycoplasmal pneumonia in pigs after experimental challenge with two field isolates. Vet Microbiol. 2012;161:186-95.
- Galindo-Cardiel I, Ballester M, Solanes D, Nofrarías M, López-Soria S, Argilaguet JM, et al. Standardization of pathological investigations in the framework of experimental ASFV infections. Virus Res. 2013;173:180-90.
